# Chronic obstructive pulmonary disease burden, grades and erythrocytosis at a tertiary hospital in western Uganda

**DOI:** 10.1186/s12890-024-02944-8

**Published:** 2024-03-06

**Authors:** Amon Banturaki, Dalton Kambale Munyambalu, Dickson Kajoba, Verah Bella Onchoke, Alina Peris, Prosper Ryamugwiza, Jacinto Amandua, Kingsley Akaba

**Affiliations:** 1 Department of Internal Medicine, Kampala International University-Teaching Hospital, P.O. BOX 71, Ishaka- Bushenyi, Uganda; 2 Department of Paediatrics and Child Health, Kampala International University-Teaching Hospital, Ishaka- Bushenyi, Uganda

**Keywords:** Chronic obstructive pulmonary disease, Erythrocytosis, Biomass fuel, Smoking

## Abstract

**Background:**

Chronic obstructive pulmonary disease (COPD) is the third leading cause of death worldwide among people over 40 years of age, and erythrocytosis is one of the major complications associated with increased mortality among COPD patients. The study aimed to determine the proportion of COPD, associated factors, and the burden of erythrocytosis among COPD participants.

**Methods and materials:**

A descriptive cross-sectional study design was used. A consecutive sampling technique was used to obtain study participants at the Fort Portal Regional Referral Hospital outpatient clinic. Focused history and physical examination were carried out to select eligible participants. Participants were screened using the COPD population screener for spirometry after consenting to participate. The study enrolled all adults at risk of having COPD based on the COPD population screener and able to undergo spirometry. Spirometry was carried out according to the Global Chronic Obstructive Lung Disease and European Respiratory Society guidelines, and haemoglobin concentration was measured.

**Results:**

One hundred eighty participants were enrolled in the study, most of whom were females.

The modal and mean age of participants was 60 years with 139 (77.2%) females and primary as the highest education level 149(82.8%). The proportion of COPD was 25% (45) [95% CI 18.9 – 32] and highest among females (68.9%) and those aged 60 years and above (70%). The combined COPD assessment tool groups had a proportion of 55.6%, 37.8%, 4.4%, and 2.2% for groups A, B, C, and D, respectively. Age < 50 years was protective against COPD, while for every additional year of smoking, there was an associated 6.5% increased risk compared to the general population. Additionally, the proportion of erythrocytosis among COPD participants was 6.7%.

**Conclusions and recommendations:**

There was a high proportion of COPD among study participants (25%), with a 6.7% proportion of erythrocytosis. We recommend a complete blood count for every patient in groups C and D of the ABCD COPD GOLD groups.

## Introduction

### Background

Chronic obstructive pulmonary disease (COPD) affects people of all age groups, races, and continents [1]. Several studies reporting the prevalence of the disease have been found to underestimate or overestimate the figures because they did not perform spirometry [2]. Among all causes of death, chronic obstructive pulmonary disease (COPD) is the third most common [3]. Nearly 80% of COPD-related fatalities, including those in the majority of African nations, are thought to occur in low- and middle-income countries due to exposure to risk factors [4, 5].

Africa is facing an increasing burden of non-communicable diseases, and COPD is among them [6, 7], with an overall prevalence of 1.568% in Sub-Saharan Africa [8]. There are variations in COPD prevalence depending on where one resides, even within the same country. In Uganda, the rural and urban COPD prevalence rates are 6.1% and 1.5%, respectively [9].

COPD is associated with hypoxemia, resulting in complications such as pulmonary hypertension, erythrocytosis, neurocognitive dysfunction, and systemic inflammation. These eventually contribute to other complications, such as acute exacerbations and cardiovascular disorders. Some patients may only present during complications and the associated poor prognosis [10, 11].

Erythrocytosis as a complication of COPD is diagnosed following findings of haemoglobin (Hb) concentration of Hb > 16.5 g/dl or hematocrit (HCT) > 49% in males and Hb > 16 g/dl or HCT > 48% in females [12]. It is also known as acquired erythrocytosis. It arises from high levels of erythropoietin as a result of chronic hypoxemia [13]. Other causes of secondary acquired erythrocytosis include cardiac disease or high altitude, hypoxia in the kidney such as in renal artery stenosis, and tumours secreting erythropoietin, e.g., renal cell cancer, hepatocellular carcinoma, and cerebellar haemangioblastoma [14].

In COPD, 20% of the patients are estimated to have erythrocytosis [15]. Erythrocytosis is attributed to the imbalance between hypoxia-induced erythropoiesis stimulation and inflammation-induced erythropoiesis depression [16]. It results in a high level of red blood cells, together with associated complications such as pulmonary hypertension and pulmonary embolism [17]. Patients with COPD have a worse prognosis when their mean pulmonary arterial pressure is roughly 19 mm Hg or more [18].

Erythrocytosis is greatly complicated by the underlying conditions in patients with COPD, such as endothelial dysfunction, coagulopathy, hypoxic vasoconstriction, emphysema-induced loss of the pulmonary capillary bed, and smoking-induced inflammatory infiltration [19].

### Objectives

Our study aimed to determine the proportions of COPD and erythrocytosis among COPD patients attending the outpatient department at Fort Portal Regional Referral Hospital.

## Methods

### Study design

The study was cross-sectional. It was carried out at the Fort Portal Regional Referral Hospital outpatient department in western Uganda between July and September 2022.

### Study site

The study took place at the Fort Portal Regional Referral Hospital (FRRH) in Uganda's Kabarole District. FRRH is a regional hospital serving the districts of Bundibugyo, Kabarole, Kamwenge, Kasese, Ntoroko, and Kyenjojo [20]. FRRH is a 330-bed capacity hospital offering both inpatient and outpatient services for emergency and chronic care with a well-established diagnostic department. The outpatient department, however, lacks a specialized clinic for respiratory disorders.

### Study population

All adult patients attending the Fort Portal Regional Referral Hospital outpatient clinic were the study population. The population in this region is made up of mostly farmers, and the region is largely rural.

### Target population

Only those patients who were likely to have COPD as screened by the COPD population screener were targeted among patients visiting the outpatient department of Fort Portal Regional Referral Hospital.

### Selection criteria

#### Inclusion criteria

Adults (18 years and above) with a total of 5 or more on the COPD Population Screener (COPD PS) were enrolled in the study [21–23].

#### Exclusion criteria

The exclusion criteria were focused on the inability to carry out spirometry, such as critically ill patients and patients with dehydration, sepsis, and shock. Other exclusion factors included current usage of diuretics, patients with documented evidence of diseases associated with erythrocytosis, e.g., renal stenosis, brain tumours, liver tumours, cyanotic heart disease, and a history of lung resection and thoracic surgery. Additionally, systemic hypotension or severe hypertension and respiratory infections such as pulmonary tuberculosis were excluded [24].

### Recruitment and sampling

Participants for the study were recruited by consecutive sampling. The enrolment process occurred at the Fort Portal Regional Referral Hospital outpatient clinic.

All participants were informed of the study procedures, and those likely to have COPD were selected using a COPD population screener questionnaire (COPD PS). The COPD PS includes three COPD-related questions (breathlessness, productive cough, and activity limitation) as well as two other questions about smoking habits and age. This questionnaire has been validated for use as a prespirometry screening questionnaire [22, 25]. Respondents with five or more points on the COPD PS were retained and eligible to participate in the study and were further requested for consent.

Those who did not consent were excluded from the study and allowed to continue with routine outpatient department (OPD) care. The participants who consented to the study had their history taken and underwent a physical examination and measurement of oxygen saturation. The study participants were then investigated by spirometry for the diagnosis of COPD. The EasyOne Plus spirometer was used for the diagnosis of COPD.

Spirometry followed the GOLD guidelines and the European Respiratory Society (ERS) for spirometry [26]. Those with airflow limitation of FEV1/FVC ratio < 0.70 were enrolled for postbronchodilator spirometry. Bronchodilation was achieved by administering 400 μg of salbutamol. Spirometry was repeated after a 15-minute interval. Those with persistent airflow limitation, i.e., less than 12% FEV1 postbronchodilator change, were diagnosed with COPD.

Participants who were found to have COPD underwent COPD severity score and grouping using the modified Medical Research Council scores, and combined COPD assessment into groups. A summary of COPD groups as classified in the study is given below following the GOLD guidelines of 2019:

- GOLD 1—mild: FEV1 ≥ 80% predicted
- GOLD 2—moderate: 50% ≤ FEV1 < 80% predicted
- GOLD 3—severe: 30% ≤ FEV1 < 50% predicted
- GOLD 4—very severe: FEV1 < 30% predicted
- Group A: minimal risk (0–1 exacerbation per year, no hospitalization required) and fewer symptoms (mMRC 0–1 or CAT < 10), GOLD 1–2 (mild or moderate airflow limitation)
- Group B: minimal risk (0–1 exacerbation per year, no hospitalization required) and more symptoms (mMRC ≥ 2 or CAT ≥ 10), GOLD 1–2 (mild or moderate airflow limitation)
- Group C: high risk (≥ 2 exacerbations per year, or one or more necessitating hospitalization) and fewer symptoms (mMRC 0–1 or CAT < 10), GOLD 3–4 (severe or very severe airflow limitation)
- Group D: high risk (≥ 2 exacerbations per year, or one or more necessitating hospitalization) and more symptoms (mMRC ≥ 2 or CAT ≥ 10), GOLD 3–4 (severe or very severe airflow limitation)

These patients further had their blood samples (2 ml) withdrawn aseptically, and haemoglobin estimation was performed using the Mission plus haemoglobin metre. The procedure was carried out by inserting a test strip into the haemoglobin metre. Ten microliters of a fresh whole blood specimen were collected using a capillary tube and dropped into the centre hole of the specimen application area of the test cartridge of the haemoglobin meter.

### Sample size determination

The sample size was determined using the Kish Leslie formula, giving a sample size of 167 participants [27].

*n* = [Z^2^P(1-P)]/d2, where n = size of the sample, P = anticipated prevalence considering a prevalence of 12.4% for COPD in Uganda [9, 28], Z = Z statistic for a level of confidence (1.96), and d = precision (0.05).

### Data processing and analysis

Data were collected using a close-ended structured questionnaire, entered into Microsoft Excel 2013 and then exported to Statistical Package for Social Science (SPSS) IBM version 28. The data analysis was done by logistic regression at bivariate and multivariate levels.

## Results

The study enrolled a total of 180 participants among the 311 adult patients attending care at Fort Portal Regional Referral Hospital OPD during the study period of June-August 2022. The participant profile is shown below:

### Baseline characteristics of the study characteristics

The majority of the study participants were females, 139 (77.2%), with a mean age of 60 (SD 14.310, skewness 0.077) and a modal age of 60 years. The majority had a maximum level of education of up to primary level 149(82.8%) and overweight or obese 100 (55.6%). Firewood was the major source of biomass burning, 159 (88.3%), with 44 (24.4%) having a history of smoking and 59 (32.8%) having a productive cough. The mean years of smoking and oxygen saturation of the participants were 3.55 years and 96.31%, respectively. This is shown in Table 1 below. The data presented were calculated as percentages of the 180 participants.

**Table 1:** Baseline characteristics of the study participants (*n* = 180)

Variable	Frequency (%)
Age (years)	
< 50	55 (30.6)
50–59	30 (16.7)
60–69	48 (26.7)
≥ 70	47 (26.1)
Sex	
Female	139 (77.2)
Male	41 (22.8)
Level of education	
At most Primary	149 (82.8)
Secondary	20 (11.1)
Tertiary	11 (6.1)
Body Mass Index (kg/m^2^)	
Undernourished	11 (6.1)
Normal	69 (38.3)
Overweight	58 (32.2)
Obese	42 (23.3)
Biomass burning	
Charcoal	17 (9.4)
Kerosene	4 (2.2)
Firewood	159 (88.3)
Housing ventilation	
Poor	57 (31.7)
Good	123 (68.3)
History of smoking tobacco	
Yes	44 (24.4)
No	136 (75.6)
Productive cough	
Yes	59 (32.8)
No	121 (67.2)
Wheezing	
Yes	49 (27.2)
No	131 (72.8)

#### Proportion of COPD

**Fig. 1:**
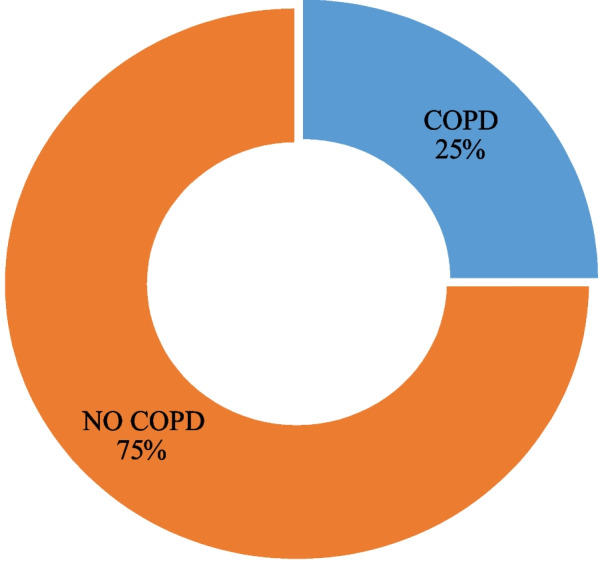
A pie chart showing the proportion of COPD among the study participants

The proportion of COPD among the study participants was 25% (45). This is shown in Figure 1 below.

#### Severity of COPD

Many of the study participants were categorized as Group A (55.6%) and B (37.8%), and only 1 participant was categorized as Group D (2.2%). This is shown in Table 2 below.

**Table 2:** A table showing the severity score of COPD using the modified Medical Research Council scores, and combined COPD assessment to groups

COPD related characteristics	
Variable	Frequency, n (%)
Modified Medical Research Council score	
Grade 0—Breathless with strenuous exercise	3 (6.7)
Grade 1—Breathless on hurrying on level ground	26 (57.8)
Grade 2—Walks slower due to breathlessness	13 (28.9)
Grade 3—Stops for breath after walking for a few minutes	2 (4.4)
Grade 4—Too breathless to leave the house or breathless on dressing	1 (2.2)
Combined COPD Assessment tool groups	
Group A	25 (55.6)
Group B	17 (37.8)
Group C	2 (4.4)
Group D	1 (2.2)

Factors associated with COPD among the study participants.

At the bivariate level, there was a statistically significant difference in mean oxygen saturation between participants with COPD and those without COPD (F-9.04, *P* value 0.003), as well as participants with COPD and those without in the mean years of smoking tobacco, and this difference was statistically significant. (F- 21.232, *P* value 0.000). Furthermore, age, history of smoking, and the use of kerosene for biomass burning were significantly associated with COPD, with a *P* value less than 0.05. Further analysis at a multivariable level found that age less than 50 years and duration of smoking were significantly associated with COPD. An age of < 50 years was associated with a 94.8% (AOR 0.052 (0.010-0.277), P 0.001) reduced chance of having COPD compared to those above 70 years, while an additional year of smoking was associated with a 6.5% (AOR 1.065 (1.013-1.119), P 0.014) increased risk of acquiring COPD compared to the general population. This is as shown in Tables 3, 4, and 5.

**Table 3:** Relationship between continuous variables and COPD by ANOVA

Variable	Outcome	Mean	Significance
Mean years of smoking tobacco	COPD—Yes	8.93	0.000
	COPD—No	1.76	
	Total	3.55	
Mean oxygen saturation	COPD—Yes	95.42	0.003
	COPD—No	96.31	
	Total	96.31

**Table 4:** Bivariable analysis of factors associated with COPD among study participants

Variable	Chronic obstructive airway disease		COR (95% C.I)	*P* value
	**No (%)**	**Yes (%)**		
Age groups				
< 50 years	53(39.3)	2(4.4)	0.04(0.01–0.18)	0.000
50 to 59 years	23(17.0)	7(15.6)	0.32(0.11–0.88)	0.028
60 to 69 years	35(25.9)	13(18.9)	0.39(0.17–0.91)	0.030
≥ 70 Years	24(17.8)	23(51.1)	1	
Sex				
Female	108(80.0)	31(68.9)	1	
Male	27(20.0)	14(31.1)	0.55(0.26–1.18)	0.124
Level of education				
Nonformal	35(25.9)	18(40.0)	5.14(0.61–43.40)	0.132
Primary	72(53.3)	24(53.3)	3.33(0.41–27.41)	0.263
Secondary	18(13.3)	2(4.4)	1.11(0.09–13.84)	0.935
Tertiary	10(7.4)	1(2.2)	1	
Body Mass Index (kg/m^2^)				
Undernourished	7(5.2)	4(8.9)		
Normal	50(37.0)	20(44.4)	0.7(0.18–2.66) 0.600	
Overweight/obese	78(57.8)	21(46.7)	0.47(0.13–1.76)	0.264
Biomass burning				
Charcoal	14(10.4)	3(6.7)	1	
Kerosene	1(0.7)	3(6.7)	14.0(1.06–185.50)	0.045
Firewood	120(88.9)	39(86.7)	1.52(0.41–5.56)	0.529
Housing ventilation				
Poor	41(30.4)	16(35.6)	1	
Good	94(69.6)	29(64.4)	0.79(0.39–1.61)	0.52
History of smoking tobacco				
Yes	25(18.5)	19(42.2)	1	
No	110(81.5)	26(57.8)	0.03(0.15–0.65)	0.001
Productive cough				
Yes	41(30.4)	18(40.0)	1	
No	94(69.6)	27(60.0)	0.65(0.33–1.32)	0.233
Wheezing				
Yes	34(25.2)	15(33.3)	1	
No	101(74.8)	30(66.7)	0.67(0.32–1.40)	0.288

**Table 5:** Multivariable analysis of the factors associated with COPD among study participants

Variable	AOR	95% C. I		*P* value
		Lower	Upper	
Age groups				
< 50 years	0.052	0.010	0.277	0.001
50 to 59 years	0.410	0.131	1.282	0.125
60 to 69 years	0.473	0.179	1.252	0.132
≥ 70 Years	Ref			
History of smoking tobacco				
Yes	Ref			
No	0.610	0.189	1.971	0.408
Biomass burning				
Charcoal	Ref			
Kerosene	18.835	0.799	444.224	0.069
Firewood	0.990	0.225	4.366	0.990
Oxygen saturation	0.876	0.742	1.035	0.120
Years Smoked	1.065	1.013	1.119	0.014

#### The burden of erythrocytosis among participants with COPD

**Fig. 2:**
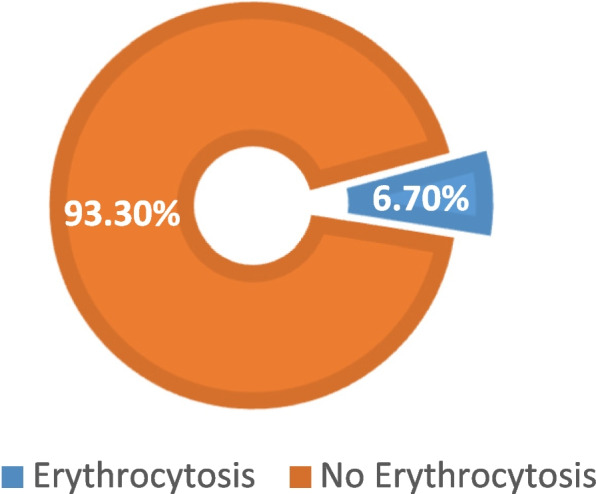
Shows the proportion of erythrocytosis in chronic obstructive pulmonary disease (COPD) study participants (*n* = 45). All participants diagnosed with COPD in the study were screened by a COPD population screener, and after that, spirometry was done to confirm the diagnosis of COPD. The participants had their haemoglobin concentration estimated. All participants with a haemoglobin level of 16.5 g/dL or higher were considered to have erythrocytosis. We found three participants had erythrocytosis out of the 45 participants who had COPD

Of the 45 participants with COPD, 3 had erythrocytosis (all of which (100%) belonged to COPD Groups C, and D), which gives a proportion of 6.7%. This is as shown in Figure 2 below:

## Discussion

**Fig. 3:**
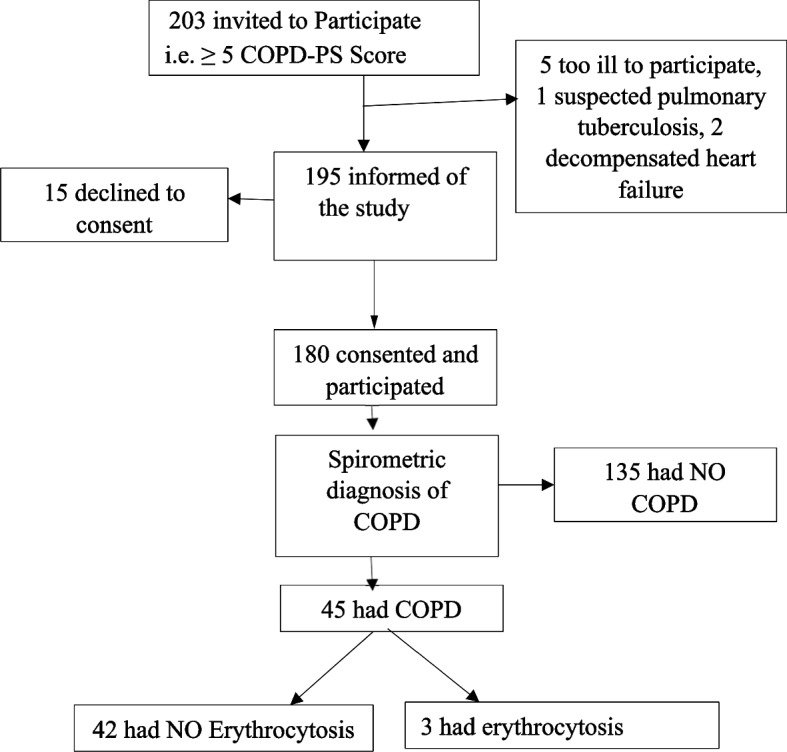
Participant enrollment flow chart

Uganda, among other sub-Saharan African countries, battles the burden of chronic obstructive pulmonary disease. In our study, the proportion of COPD among the study participants was 25% [95% CI 18.9 – 32]. This reflects an enormous burden of the disease in the region. This can be attributed to the screening process employed in our study, which used the COPD population screening tool. The screening encouraged the involvement of participants who had symptoms (Figure 3) and, thus, a higher likelihood of individuals having COPD participating in the study. The high proportion may also depict the actual high proportion of COPD in the district of Kabarole since air pollution has also been reported in the district where the study participants live with air pollution particles from biomass exceeding the WHO air quality limits [29]. A study carried out in southwestern Uganda found a low prevalence of COPD at 2% in 2019 [30], while the study in central Uganda had a proportion of 6.1% [9]. This could be because it was a community study compared to the current study, which was hospital-based. Additionally, the current study used participants who were highly at risk of having COPD, as determined by the COPD population screener.

Regarding the COPD classification, the majority of the study participants belonged to the group of mild to moderate COPD according to the COPD combined assessment tool. Among the 45 COPD participants, only 3 (6.6%) belonged to groups C and D of the combined assessment tool. This presentation is similar to a study performed in India, where groups A and B had higher numbers than groups C and D [31]. This means that the data described does not represent severe forms of COPD. Participants with COPD may have protective factors preventing and delaying progression to severe forms of COPD, such as patient education for smoking cessation, vaccination against pneumococcal infections and influenza, and minimizing exposure to noxious gases and particles, such as using face masks in dusty workplaces [9]. In our study, 123 (68.3%) participants had good house ventilation, and 136 (75.6%) had no history of cigarette smoking. These participants' characteristics could have contributed to the low number of cases of severe COPD.

The study found that participants 50 years and below had a 0.052 reduced chance of having COPD compared to those above 70 years. This reflects the fact that the risk of COPD increases with age, as it has been observed [32] that COPD is 2-3 times higher in people above 60 years of age than in younger people. Furthermore, the changes that occur in the lungs with normal ageing, such as reduced lung function and associated breathlessness, increased air trapping, reduction in lung elastic recoil, and chest wall compliance, are present in COPD-affected lungs and may also enhance the susceptibility to COPD exacerbations [32, 33]. The findings in this study could be attributed to the risk factors among the study participants leading to older-onset versus early-onset COPD [34, 35].

For every additional year of smoking, the odds of COPD increase by 1.065, according to our study, which suggests the contribution of smoking to the observed COPD. Some of the facts include that smoking damages the air sacs, airway, and lung mucosa and is also responsible for triggering COPD flare-ups [35]. Findings from the COPDGene study found that smoking duration is more significantly connected with COPD than the sum of pack years because there is a more significant airflow obstruction for smoking duration than for cigarette/day and park years. Second, it is more difficult to determine how many cigarettes one smokes per day [36]. A 25-year follow-up study of the general population in Copenhagen, Denmark, established a 25% and beyond absolute risk of developing COPD among continued smokers [37], with even low-rate smokers having an elevated chance of developing lung disease in the future [38]. Thus, there are no safe limits for the effect of smoking intensity on lung disease risk, making each added year a risk factor for COPD [39].

Erythrocytosis was found in 6.7% [95% CI 1.4-18.3] of the 45 COPD participants. The findings were similar to those of a study in China in which 886 COPD patients were assessed for erythrocytosis, with a prevalence of 6.4% [39]. However, a study carried out in Pakistan had a higher prevalence of 10.8% [40], but the study findings were higher than those observed in Egypt (2.4%) [40]. The differences in prevalence may be due to differences in the inclusion criteria used by these studies and the risk factors supporting the development of erythrocytosis. The progression of erythrocytosis may be multifactorial and thus needs to be studied in various populations. Male sex, current smoking, poor carbon monoxide diffusing capacity (DLCO), and severe hypoxemia are all linked to an elevated risk for secondary erythrocytosis [41]. However, erythrocytosis among COPD patients increases the likelihood of acute respiratory failure [42], pulmonary embolism (PE), and death [43].

## Limitations

The study participants found to have erythrocytosis were not investigated for other causes, such as polycythemia vera. Other limitations included: the study being cross-sectional without follow-up of participants, a small sample size, and the use of data for both tobacco-associated and biomass-associated COPD in the same study.

## Conclusions and recommendations

There was a high proportion of erythrocytosis, 6.7% among participants with COPD and 100% among those with severe COPD. Hence, a complete blood count should be performed on patients with severe COPD at diagnosis and follow-up. Furthermore, an algorithm should be developed in the management of patients with severe COPD to mitigate against complications associated with erythrocytosis in this group of patients.

## Data Availability

The datasets used and/ or analysed during the current study are available from the corresponding author on reasonable request.
